# Comparative and Evolutionary Physiology of Vasopressin/ Oxytocin-Type Neuropeptide Signaling in Invertebrates

**DOI:** 10.3389/fendo.2020.00225

**Published:** 2020-04-17

**Authors:** Esther A. Odekunle, Maurice R. Elphick

**Affiliations:** School of Biological & Chemical Sciences, Queen Mary University of London, London, United Kingdom

**Keywords:** vasopressin, oxytocin, neuropeptide, receptors, diuresis, reproduction, social behavior, feeding

## Abstract

The identification of structurally related hypothalamic hormones that regulate blood pressure and diuresis (vasopressin, VP; CYFQNCPRG-NH_2_) or lactation and uterine contraction (oxytocin, OT; CYIQNCPLG-NH_2_) was a major advance in neuroendocrinology, recognized in the award of the Nobel Prize for Chemistry in 1955. Furthermore, the discovery of central actions of VP and OT as regulators of reproductive and social behavior in humans and other mammals has broadened interest in these neuropeptides beyond physiology into psychology. VP/OT-type neuropeptides and their G-protein coupled receptors originated in a common ancestor of the Bilateria (Urbilateria), with invertebrates typically having a single VP/OT-type neuropeptide and cognate receptor. Gene/genome duplications followed by gene loss gave rise to variety in the number of VP/OT-type neuropeptides and receptors in different vertebrate lineages. Recent advances in comparative transcriptomics/genomics have enabled discovery of VP/OT-type neuropeptides in an ever-growing diversity of invertebrate taxa, providing new opportunities to gain insights into the evolution of VP/OT-type neuropeptide function in the Bilateria. Here we review the comparative physiology of VP/OT-type neuropeptides in invertebrates, with roles in regulation of reproduction, feeding, and water/salt homeostasis emerging as common themes. For example, we highlight recent reports of roles in regulation of oocyte maturation in the sea-squirt *Ciona intestinalis*, extraoral feeding behavior in the starfish *Asterias rubens* and energy status and dessication resistance in ants. Thus, VP/OT-type neuropeptides are pleiotropic regulators of physiological processes, with evolutionarily conserved roles that can be traced back to Urbilateria. To gain a deeper understanding of the evolution of VP/OT-type neuropeptide function it may be necessary to not only determine the actions of the peptides but also to characterize the transcriptomic/proteomic/metabolomic profiles of cells expressing VP/OT-type precursors and/or VP/OT-type receptors within the framework of anatomically and functionally identified neuronal networks. Furthermore, investigation of VP/OT-type neuropeptide function in a wider range of invertebrate species is now needed if we are to determine how and when this ancient signaling system was recruited to regulate diverse physiological and behavioral processes in different branches of animal phylogeny and in contrasting environmental contexts.

## Introduction

### The Discovery and Functional Characterization of Vasopressin/Oxytocin-Type Neuropeptide Signaling in Mammals

The importance of the structural characterization of the pituitary neurohormones vasopressin (VP) and oxytocin (OT) was recognized in the award of the 1955 Nobel Prize for chemistry to Vincent du Vigneaud. This was the culmination of a programme of research dating back to 1895, when Oliver and Schafer reported that a substance in extracts of the pituitary gland elevates blood pressure when injected intravenously into dogs (1). It was established later that this vasopressor originates from the posterior pituitary or neurohypophysis (2). Dale then reported that a neurohypophysial substance triggers uterine contraction (3, 4) and other bioactivities of pituitary extracts were discovered, including stimulation of lactation (5, 6) and antidiuresis (7). Purification of these bioactive components of pituitary gland extracts revealed that the vasopressor and antidiuretic activity could be attributed to one substance (VP) and the uterotonic and lactation-promoting activity could be attributed to another substance (OT) (8). In the 1950s the amino acid sequences and secondary structures of VP (9–11) and OT (12, 13) were determined and both peptides were chemically synthesized (14, 15). This revealed that VP (CYFQNCPRG-NH_2_) and OT (CYIQNCPLG-NH_2_) are structurally very similar, with only two amino acids differing, indicative of a common evolutionary origin. Furthermore, both peptides have a disulphide bridge between the cysteine residues at positions one and six, which is a conserved feature of all VP/OT-type peptides that have been identified subsequently (Table 1).

**Table 1:** Amino acid sequences of vasopressin/oxytocin-type neuropeptides in species that belong to a variety of bilaterian phyla/sub-phyla.

**Phylum/** **Sub-phylum**	**Species (selected peptide** **names)**	**Sequence**
Vertebrata	*Homo sapiens* (vasopressin)	CYFQNCPRG-NH_2_
	*Homo sapiens* (oxytocin)	CYIQNCPLG-NH_2_
	*Lethenteron japonicum* (vasotocin)	CYIQNCPRG-NH_2_
Urochordata	*Styela plicata*	CYISDCPNSRFWST-NH_2_
	*Ciona intestinalis*	CFFRDCSNMDWYR
Cephalochordata	*Branchiostoma floridae*	CYIINCPRG-NH_2_
Hemichordata	*Saccoglossus kowalevskii*	CFISDCARG-NH_2_
Echinodermata	*Strongylocentrotus purpuratus*	CFISNCPKG-NH_2_
	*Apostichopus japonicus*	CFITNCPLG-NH_2_
	*Asterias rubens*	CLVQDCPEG-NH_2_
	*Ophionotus victoriae*	CLVSDCPEG-NH_2_
Xenacoelomorpha	*Xenoturbella bocki*	CLVQGCPIG-NH_2_
	*Xenoturbella profunda*	CLVQGCPIG-NH_2_
	*Ascoparia* sp	CVIVACPRG-NH_2_
Arthropoda	*Locusta migratoria*	CLITNCPRG-NH_2_
	*Tribolium casteneum*	CLITNCPRG-NH_2_
	*Atta cephalotes*	CLITNCPRG-NH_2_
	*Camponotus floridanus*	CLIVNCPRG-NH_2_
	*Harpegnathos saltator*	CLITNCPRG-NH_2_
	*Lasius niger*	CLITNCPRG-NH_2_
	*Lasius neglectus*	CLITNCPRG-NH_2_
	*Cancer borealis*	CFITNCPPG-NH_2_
	*Portunus pelagicus*	CFITNCPPG-NH_2_
	*Strigamia maritima*	CYITNCPPG-NH_2_
	*Sarcoptes scabiei*	CFITNCPPA-NH_2_
Nematoda	*Caenorhabditis elegans*	CFLNSCPYRRY-NH_2_
Tardigrada	*Hypsibius dujardini*	CFVTNCPPG-NH_2_
	*Ramazzottius variernatus*	CFVTNCPPG-NH_2_
Mollusca	*Conus striatus*	CIIRNCPRG-NH_2_
	*Conus geographus*	CFIRNCPKG-NH_2_
	*Lymnaea stagnalis*	CFIRNCPKG-NH_2_
	*Octopus vulgaris* (cephalotocin)	CYFRNCPIG-NH_2_
	*Octopus vulgaris* (octopressin)	CFWTSCPIG-NH_2_
	*Sepia officinalis* (sepiatocin)	CFWTTCPIG-NH_2_
	*Sepia officinalis* (pro-sepiatocin)	CFFRNCPPG-NH_2_
	*Mizuhopecten yessoensis*	CFIRNCPPG-NH_2_
Annelida	*Erpobdella octoculata*	CFIRNCPKG-NH_2_
	*Eisenia fetida*	CFVRNCPTG-NH_2_
	*Platynereis dumerilii*	CFVRNCPPG-NH_2_

*Insights into the physiological roles of many of these neuropeptides have been obtained and are discussed in this review*.

OT and VP are derived from precursor proteins that contain the cysteine-rich proteins neurophysin-I and neurophysin-II, respectively. Neurophysins bind OT or VP and are required for normal targeting of these neuropeptides to the regulated secretory pathway (16). Three G-protein coupled receptors (GPCRs) mediate the effects of VP (V1aR, V1bR, V2R) and a single GPCR mediates the effects of OT (OTR) (17). Consistent with the actions of OT as a regulator of lactation and uterine tone, OTR is expressed in the mammary glands and uterus, respectively. Consistent with the actions of VP as a vasopressor and antidiuretic, V1aR is expressed in vascular smooth muscle and V2R is expressed in the kidney. However, all four receptors are also expressed in other tissues/organs and perhaps most notably OTR, V1aR, and V1bR are widely expressed in the brain (18). Investigation of the functional significance of VP/OT-type receptor expression in the brain has revealed that VP/OT-type signaling regulates reproductive and social behavior in humans and other mammals (19), discoveries that have broadened interest in VP/OT-type neuropeptides beyond physiology into psychology.

Whilst VP and OT are now perhaps best known for their roles in regulation of reproductive and social behavior, other brain-associated actions in mammals have also been discovered; for example, intracerebroventricular injection of OT inhibits food and fluid intake in rats (20). Thus, as with other neuropeptides, both VP and OT are pleiotropic in their actions and our understanding of their physiological roles in mammals requires an integrative physiological and behavioral perspective, as discussed recently by Leng and Russell (21). Furthermore, to understand not only what VP and OT do in mammals but also why they do what they do, an evolutionary and comparative perspective is needed. Accordingly, there is a rich history of research on VP/OT-type signaling in non-mammalian vertebrates. This has been reviewed extensively elsewhere but a brief overview is presented below to serve as a prelude to the main theme of this review, which focuses on research investigating the physiological roles of VP/OT-type neuropeptides in invertebrates.

### Evolution and Comparative Physiology of VP/OT-Type Neuropeptide Signaling in Non-mammalian Vertebrates

Analysis of genome sequence data from non-mammalian vertebrates has enabled reconstruction of the evolutionary history of VP/OT-type signaling in the vertebrate lineage. The most primitive extant vertebrates are the jawless fish (lampreys, hagfish; Agnatha) and analysis of the genome sequence of the lamprey *Lethenteron japonicum* revealed that it has a single gene encoding a VP/OT-type neuropeptide (“vasotocin;” CYIQNCPRG-NH_2_) (22) (Table 1). This contrasts with jawed vertebrates (gnathostomes) that typically have two genes encoding VP/OT-type neuropeptides—one that is an ortholog of the mammalian VP gene and another that is an ortholog of the mammalian OT gene. Thus, it has been inferred that the VP-type and OT-type genes originated by tandem duplication of a single VP/OT-type gene in a common ancestor of the gnathostomes (22). Furthermore, this was preceded in a common ancestor of the vertebrates by a gene duplication that gave rise to two genes encoding VP/OT-type receptors. Then two rounds of whole-genome duplication during early vertebrate evolution gave rise to eight genes encoding VP/OT-type receptors, with subsequent lineage-specific gene loss and additional gene/genome duplication events resulting in the variable numbers of VP/OT-type precursor genes and VP/OT-type receptor genes that are found in extant vertebrates (23, 24).

Prior to the genome-sequencing era, a variety of VP/OT-type neuropeptides were identified in non-mammalian vertebrates. With the benefit of hindsight, the nomenclature that was chosen for VP/OT-type neuropeptides in non-mammalian vertebrates is potentially confusing. For example, the name “vasotocin” was given to peptides that are orthologs of VP and the names “mesotocin” and “isotocin” were given to peptides that are orthologs of OT (25). Nevertheless, the discovery of these peptides in non-mammalian vertebrates was very important because it enabled analysis of their physiological roles. For example, in teleost fish vasotocin has been found to have VP-like roles in osmoregulation and cardiovascular physiology as well as OT-like roles in regulation of reproduction (26). Furthermore, central administration of isotocin in goldfish inhibits food intake (27), consistent with the anorexigenic effect of OT in mammals (20). However, a detailed review of the physiological roles of VP/OT-type neuropeptides in non-mammalian vertebrates is beyond the scope of this article, and for this topic we refer readers to other reviews (25, 28–30).

### Discovery of VP/OT-Type Neuropeptide Signaling in Invertebrates

Immunocytochemical evidence that VP-like neuropeptides occur in invertebrates was first reported in the late 1970s. Thus, two cells immunoreactive with antibodies to VP and to neurophysin II were identified in the suboesophageal ganglion of the locust *Locusta migratoria* (31). Subsequently, a VP-like peptide (CLITNCPRG-NH_2_) was purified from extracts of *L. migratoria* suboesophageal ganglia and, interestingly, both a monomeric peptide (F1) and an anti-parallel dimer of the F1 peptide (F2) were identified (32) (Table 1). In parallel with research on insects, the existence of VP-like substances in molluscan species was also reported (33, 34). Then in 1987, VP-like peptides named Lys-conopressin G (CFIRNCPKG-NH_2_) and Arg-conopressin S (CIIRNCPRG-NH_2_) were purified from the venom of the cone snails *Conus geographus* and *Conus striatus*, respectively, and sequenced (35) (Table 1). Thus, the discovery of VP-like peptides in both insect and molluscan species in 1987 provided the first definitive molecular evidence of the occurrence of VP/OT-type peptides in invertebrates, demonstrating the evolutionary antiquity of this neuropeptide family. Accordingly, the presence of VP-like immunoreactivity in insects, molluscs and a variety of other invertebrates was reported the following year (36).

A VP-like peptide identical in structure to Lys-conopressin G was purified from extracts of the pond snail *Lymnaea stagnalis* (Table 1) and, importantly, cloning and sequencing of the gene encoding the precursor of this peptide revealed evolutionary conservation of protein structure. Thus, as in vertebrate VP/OT-type precursors, the neuropeptide is located immediately after an N-terminal signal peptide and the C-terminal region of the precursor comprises a neurophysin domain (37). Furthermore, a G-protein coupled receptor that shares sequence similarity with vertebrate VP/OT-type receptors and that mediates the effects of Lys-conopressin in *L. stagnalis* was identified, revealing evolutionary conservation of an ancient neuropeptide-receptor signaling pathway (38).

The first genome sequence of an animal species was reported in 1998 with the sequencing of the genome of the nematode *Caenorhabditis elegans* (39) and subsequently genes encoding the VP/OT-type neuropeptide nematocin and two cognate receptors were identified in this species (40–42). Likewise, as the genomes and/or transcriptomes of many other invertebrate species have been sequenced over the last two decades, genes/transcripts encoding VP/OT-type precursors and receptors have been identified in an ever-increasing variety of invertebrate taxa, as discussed in more detail below. It is in this context that here we go on to discuss in detail what is known about the occurrence, characteristics and physiological roles of VP/OT-type signaling in invertebrate taxa. In doing so, we build upon, complement, extend and update several related review articles that have been published previously (30, 43–47).

## Comparative Physiology of VP/OT-Type Neuropeptide Signaling in Invertebrates

**Figure 1:**
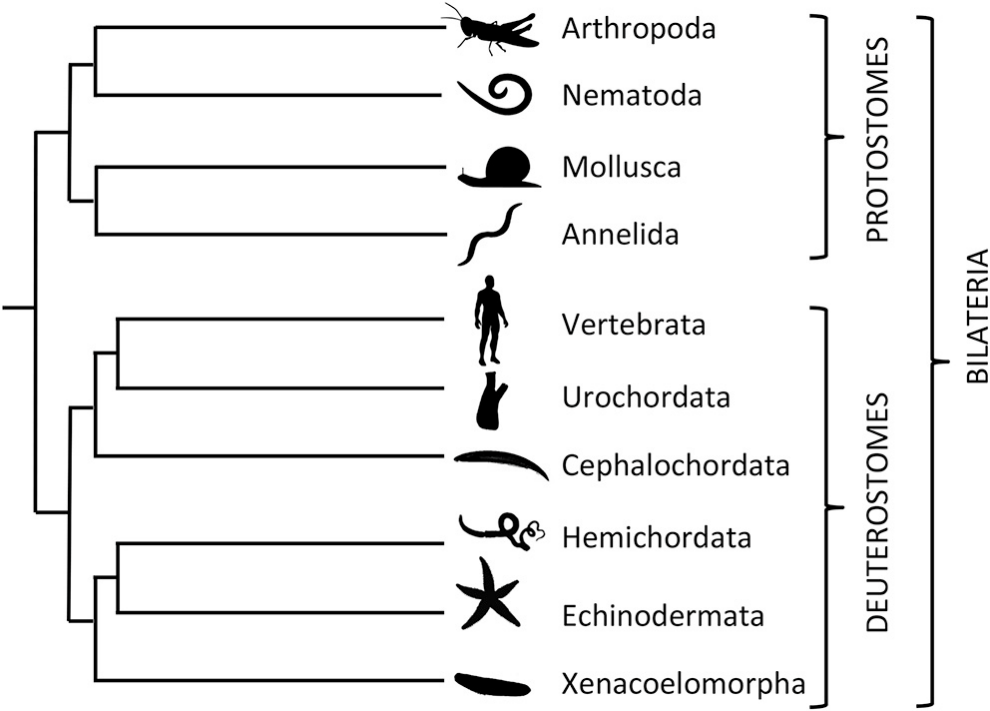
Phylogenetic tree showing relationships of selected bilaterian phyla and sub-phyla discussed in this review. The Bilateria comprise two main clades: The protostomes, which include ecdysozoan phyla—Arthropoda (e.g., locust *Locusta migratoria* and fruit fly *Drosophila melanogaster*) and Nematoda (e.g., *Caenorhabditis elegans*)—and spiralian phyla—Mollusca (e.g., pond snail *Lymnaea stagnalis* and sea slug *Aplysia californica*) and Annelida (e.g., earthworm *Eisenia fetida* and ragworm *Platynereis dumerilii*). The deuterostomes, which include three chordate sub-phyla—Vertebrata (e.g., *Homo sapiens*), Urochordata (e.g., sea-squirt *Ciona intestinalis*) and Cephalochordata (e.g., lancelet *Branchiostoma floridae*)—and the xenambulacrarian phyla—Hemichordata (e.g., acorn worm *Saccoglossus kowalevskii*), Echinodermata (e.g., starfish *Asterias rubens*), and Xenacoelomorpha (e.g., *Xenoturbella bocki*). The branch lengths in the tree are arbitrary.

As a framework for investigation of the comparative physiology of VP/OT-type neuropeptide signaling, it is necessary to briefly introduce bilaterian phylogeny. The Bilateria comprise two major clades—the protostomes and deuterostomes. The protostomes are further sub-divided into the Ecdysozoa (e.g., arthropods, nematodes) and the Spiralia (e.g., molluscs, annelids), whilst the deuterostomes comprise vertebrates, invertebrate chordates (urochordates, cephalochordates), hemichordates and echinoderms (48) (Figure 1). In discussing the comparative physiology of VP/OT-type neuropeptide signaling below, we have arbitrarily elected to start with the ecdysozoans because it was in an insect species, the locust *L. migratoria*, that the first invertebrate insights into VP/OT-type neuropeptide structure and function were obtained (32, 49). We then progress to the spiralian protostomes before moving on to the invertebrate deuterostomes, which are of particular interest because of their close relationship with vertebrates. We discuss the phylum Xenacoelomorpha last because the phylogenetic position of this phylum is controversial, as discussed in more detail below.

### Ecdysozoa

#### Arthropoda

The purification and sequencing of a VP/OT-type neuropeptide from extracts of the suboesophageal ganglion of the locust *L. migratoria* provided definitive molecular evidence that this neuropeptide type exists in invertebrates (32). Subsequently, VP/OT-type neuropeptides have been identified in many other insects and the G-protein coupled receptors that mediate the effects of these peptides have been identified and pharmacologically characterized. Interestingly, however, VP/OT-type neuropeptide signaling has been lost in some insect lineages, including the model insect species *Drosophila melanogaster* (50).

Analysis of the distribution of VP-like immunoreactivity in the locust *L. migratoria* revealed a pair of immunostained neuronal cell bodies in the suboesophageal ganglion (31). Furthermore, use of radioimmunoassay methods revealed the presence of VP-like immunoreactivity in other regions of the locust body, including the brain, thoracic ganglia, abdominal ganglia, rectum, Malpighian tubules and hemolymph (51). Subsequently, a detailed analysis of the anatomy of the VP-like immunoreactive (VPLI) neurons of *L. migratoria*, combining use of immunohistochemistry with Lucifer Yellow or cobalt staining, revealed the processes of these neurons in the brain (optic lobe), suboesophageal ganglion, thoracic ganglia and abdominal ganglia (52). Furthermore, comparative analysis of 16 other grasshopper species revealed that the occurrence of a pair of VPLI neurons in the suboesophageal ganglion is a conserved feature of this family of insects, but with differences in the anatomy of their arborisations (53).

Insights into the potential physiological roles of the VP-type neuropeptide in locusts were first obtained with the discovery that injection of crude or purified extracts of suboesophageal ganglia causes a dose-dependent increase in the rate of dye excretion by Malpighian tubules *in vivo*. However, injection of mammalian VP had no effect on dye excretion in locusts and so it was unclear if the effect of ganglion extracts could be attributed to a VP-type peptide (54). With the isolation, sequencing and synthesis of the locust VP-type peptides F1 (monomeric peptide) and F2 (anti-parallel homodimer) (32, 49), it became possible to test the effects of these peptides on fluid secretion by Malpighian tubules *in vitro*. Interestingly, F1 was found to have no effect on urine production, whereas the anti-parallel dimer F2 appeared to have a stimulatory effect on diuresis (32). Subsequently, F1, F2 and a parallel dimer of F1 (PMd) were tested on *in vitro* preparations of Malpighian tubules from *L. migratoria* but none of the peptides affected fluid secretion (55). Thus, the proposed physiological role of a VP-type peptide as a diuretic hormone in locusts remained unproven.

With respect to a potential role in regulation of diuresis, investigation of the electrophysiological properties of the pair of VPLI neurons that synthesize the VP-type neuropeptide in *L. migratoria* revealed that the activity of these neurons is not affected by the osmolality of perfusion salines. However, spiking activity of the neurons was found to be inversely related to light intensity, with the neurons receiving input from a pair of brain descending interneurons that form part of an extra-occular photoreceptor system (56). Further characterization of the VPLI neurons revealed that cholinergic inputs maintain the spiking activity of these neurons in the dark via both muscarinic and nicotinic acetylcholine receptors (57). Furthermore, stimulation of VPLI neuron activity was found to cause a reduction in cAMP levels in the locust brain. Biochemical analysis of isolated VPLI neurons revealed that they contain F1 and both the anti-parallel (F2) and parallel (PDm) dimers of F1, but only F1 was found to inhibit a forskolin-stimulated increase in cAMP in isolated locust neural membranes (58). In conclusion, over a period of almost two decades from the 1970s to the 1990s detailed insights into the biochemistry, anatomy, and physiology of VP-type peptides produced by the pair of VPLI neurons in the suboesophageal ganglion of the locust *L. migratoria* were obtained. More recently, a partial sequence of the gene encoding the VP-type peptide in *L. migratoria* has been reported (59). Further characterization of this gene would facilitate use of gene knockdown/knockout techniques to gain new insights into the physiological roles of VP-type signaling in locusts.

Sequencing of the *D. melanogaster* genome (60) revealed that VP/OT-type signaling has been lost in this species (50), which precluded its use as a model for functional studies on this neuropeptide signaling system. Likewise, genome sequencing revealed loss of VP/OT-type signaling in other insect species, including the mosquito *Anopheles gambiae* and the honey bee *Apis mellifera* (50). However, sequencing of the genome of the red flour beetle *Tribolium casteneum* revealed the existence of a gene encoding a VP/OT-type peptide precursor in an insect species (61–63). Furthermore, the *T. casteneum* VP/OT-type neuropeptide (Table 1) was named “inotocin” and its receptor was identified and characterized pharmacologically (50). Analysis of the expression of the genes encoding inotocin and its cognate receptor using qPCR revealed that in adult animals the expression level of both genes is highest in the head, but with receptor expression also detected in hind gut/Malpighian tubules. Interestingly, a developmental analysis revealed that expression of both genes is highest during the larval stage soon after hatching (50), which may indicate that VP/OT-type signaling is physiologically more important at this stage of development than in adults. Molecular and pharmacological characterization of the VP/OT-type signaling system in *T. castenuem* was also reported by Aikins et al. showing that the monomeric VP-type peptide (F1) is more potent as a ligand for the receptor than anti-parallel (F2) and parallel (D2) dimers of F1 (64). Consistent with previous findings from locusts, immunocytochemical analysis revealed expression of the VP-type peptide in a pair of neurons located in the suboesophageal ganglion of *T. castenuem*, with processes projecting anteriorly into the brain and posteriorly into the thoracic and abdominal ganglia of the ventral nerve cord. Furthermore, investigation of the physiological roles of VP/OT-type signaling in *T. castenuem* revealed that the monomeric F1 peptide stimulates diuresis in this species (64). However, neither the monomeric peptide (F1) nor dimers of F1 (F2, D2) stimulated fluid secretion when tested on *in vitro* preparations of Malpighian tubules from the another beetle species, *Tenebrio molitor*, consistent with previous findings from locusts (55). Interestingly, F1-stimulated fluid secretion was observed when Malpighian tubules were co-incubated with the central nervous system (including neurohormone secreting glands—the corpora cardiaca and corpora allata). Therefore, the authors concluded that the VP-type peptide stimulates diuresis in beetles not by acting directly on the Malpighian tubules but by stimulating the release of a diuretic substance from neural/neurohemal tissues (64), a mechanism of action that may also apply to other insects.

Most recently, advances in our knowledge of the physiological/behavioral roles of VP-type signaling in insects have been obtained through experimental studies on ants. Analysis of genome sequence data enabled identification of genes encoding a VP-type (inotocin) precursor in three ant species—*Atta cephalotes* (leaf-cutter ant), *Camponotus floridanus* (carpenter ant) and *Harpegnathos saltator* (Indian jumping ant) (65) (Table 1). Pharmacological characterization of a VP-type receptor in the black garden ant *Lasius niger* revealed that inotocin is a potent ligand for this receptor, with an EC_50_ of 22 pM. Furthermore, it was discovered that an analog of *L. niger* inotocin (Table 1) containing a D-isomer of arginine at position 8 ([D-arg8]-inotocin) acts as a potent and selective antagonist of the human V1A receptor, providing a basis for translation of research on VP-type signaling in insects into potential clinical applications (66).

To gain insights into the physiological/behavioral roles of inotocin signaling in *L. niger*, inotocin receptor expression in the head of queen ants was measured. This revealed that expression levels were highest early in queen life when they experience crowded conditions in their mother nests before leaving to mate (67). Subsequently, a more detailed examination of the expression and physiological/behavioral roles of inotocin was reported, using the ant species *Lasius neglectus* as a model system (68) (Table 1). As reported previously in locusts (see above), inotocin expression was revealed in a pair of neurons in the suboesophageal ganglion. In addition, a detailed analysis of the expression levels of the inotocin precursor gene and the inotocin receptor gene in different developmental stages, castes and tissues/organs was reported. Furthermore, it was discovered that expression levels of both the inotocin precursor gene and the inotocin receptor gene are two-three fold higher in the summer than in the winter. Because feeding activity of ants is highest in the summer, the authors also examined gene expression with respect to feeding status and discovered that expression of the inotocin precursor gene is down regulated after a two-day starvation period. Knockdown of inotocin precursor gene expression using RNAi revealed changes in the expression of ~100 other genes, many of which were found to be associated with fat, protein and DNA metabolism. Furthermore, behavioral analysis of ants in which inotocin precursor gene expression had been knocked-down revealed increased locomotor activity and increased self-grooming in the brood chamber. Based on these findings, the authors concluded that inotocin signaling is important for regulation of energy status in association with locomotion and appetite in *Lasius neglectus* (68).

Complementary to Gruber and colleagues' detailed analysis of inotocin signaling in ant species of the genus *Lasius*, Koto et al. recently reported functional characterization of inotocin signaling in ant species of the genus *Camponotus* (69). The expression levels of the inotocin precursor gene and the inotocin receptor gene in different body parts were compared between mated queens, virgin queens, males, and workers with, for example, significantly higher levels of abdominal receptor gene expression detected in workers than in other castes. Furthermore, whole-body expression of both the precursor gene and the receptor gene were found to be upregulated as workers age and switch tasks from nursing to foraging. More specifically, inotocin precursor expression positively correlated with the overall level of activity, but not with time spent at food sources or in the nest. Consistent with previously reported findings from other insect species, immunohistochemical analysis revealed that inotocin is expressed in a pair of neurons in the suboesophageal ganglion. Furthermore, a cluster of inotocin-expressing neurons was also revealed in the protocerebrum of the brain. Analysis of inotocin receptor expression revealed high levels of transcript abundance in the fat body and use of mRNA *in situ* hybridization showed that these transcripts are specifically located in oenocytes, cells that are implicated in fatty acid and hydrocarbon metabolism. Experimental studies on *D. melanogaster* indicate that oenocytes produce cuticular hydrocarbons (CHCs) that are required for dessication resistance and pheromonal communication (70). Furthermore, the final step in CHC biosynthesis is catalyzed by the enzyme CYP4G1, which is expressed in *Camponotus* oenocytes. Therefore, inotocin signaling was investigated as a potential regulator of CYP4G1 and CHC biosynthesis in this species. Down-regulation of inotocin receptor expression was found to cause a reduction in *CYP4G1* expression and accordingly injection of inotocin receptor antagonists also caused a reduction in *CYP4G1* expression. Furthermore, injection of an inotocin receptor antagonist caused a reduction in hydrocarbon (alkane) synthesis and dessication resistance. Collectively, these findings led the authors to propose that when *Camponotus* worker ants start foraging, where there is an increased risk of dessication, inotocin signaling acts to stimulate synthesis of protective CHCs. In this context it is interesting that knockdown of inotocin expression in *L. neglectus* causes increased locomotor activity (68). Thus, inotocin signaling may act at a physiological level to stimulate synthesis of protective CHCs (*Camponotus*) and at a behavioral level to inhibit locomotor activity (*L. neglectus*) as integrated adaptive mechanisms to minimize dessication. These findings are of interest with respect to the anti-diuretic action of VP in mammals because they are suggestive of an evolutionarily ancient role of VP/OT-type signaling as a regulator of water homeostasis. What remains to be understood is the evolutionary/functional relationship between the water-preserving action of inotocin signaling in ants and the diuretic action of VP-type signaling in locusts and beetles (see above). To address this issue, experimental analysis of inotocin signaling in a wider range of insect species is now needed. Opportunities to do this have been afforded by sequencing of the transcriptomes/genomes of over 200 insect species and identification of genes encoding the inotocin precursor and/or inotocin receptor in these species. It should be noted, however, that VP/OT-type signaling has been lost in several insect lineages (71).

Currently, relatively little is known about VP/OT-type signaling in other arthropods. However, genes encoding VP/OT-type precursors and receptors have been identified in several non-insect arthropod taxa, including crustaceans, myriapods (millipedes and centipedes; e.g., *Strigamia maritima*) and chelicerates (scorpions, horseshoe crabs and mites; e.g., *Sarcoptes scabiei*, but with loss of this signaling system in spiders) (71) (Table 1). Furthermore, in several crustacean species the expression of these genes has been investigated in a functional context, as discussed below.

The stomatogastric nervous system, which controls feeding/digestion-associated processes in crustaceans, has been used as a model “simple” system in neurobiology to investigate how rhythmic motor-output is generated by a small neuronal network. Furthermore, neuropeptides have been identified that cause changes in motor output by altering synaptic connectivity within neural circuits (72). Expression of a VP/OT-type receptor has been detected in the stomatogastric ganglion of the crab *Cancer borealis* (73, 74) but the effects of the *C. borealis* VP/OT-type neuropeptide CFITNCPPG-NH_2_ (Table 1) on the stomatogastric system have yet to be investigated.

A cDNA encoding the precursor of the VP/OT-type peptide CFITNCPPG-NH_2_ in the blue swimming crab *Portunus pelagicus* (Table 1) has been sequenced and analysis of the expression of this precursor revealed transcripts in the eyestalk, brain, ventral nerve cord (VNC), intestine, gill and ovary. More specifically, transcripts were detected in oocytes and the VP/OT-type peptide in this species caused inhibition of steroid release from the ovary (75).

Expression of a VP/OT-type neuropeptide precursor in the shore crab *Carcinus maenas* has been examined in the context of ecdysis, which is regulated by release of ecdysteroid molting hormones from the endocrine Y-organs. Changes in Y-organ expression of the VP/OT-type neuropeptide precursor transcripts were detected over the molt cycle, but no changes in VP/OT-type receptor expression were observed (76).

Further studies are now needed to gain more detailed insights into the roles of VP/OT-type signaling in regulation of physiological processes in crabs and other crustaceans, with the potential for applicability in aquaculture of economically important species.

#### Nematoda

Phylogenomic analysis of the sequences of VP/OT-type precursor proteins facilitated identification of a gene encoding the VP/OT-type peptide “nematocin” in the nematode *C. elegans* (40). Subsequently, mass spectrometric analysis of extracts of *C. elegans* revealed that, by comparison with the majority of VP/OT-type neuropeptides in other taxa, nematocin has an unusual structure—CFLNSCPYRRY-NH_2_ (Table 1). Thus, in common with other VP/OT-type peptides it has a disulphide bridge between the cysteines and a C-terminal amide group, but it is a C-terminally extended 11-residue peptide. There are two nematocin receptors in *C. elegans*, NTR-1 and NTR-2. Nematocin causes a dose-dependent increase in intracellular Ca^2+^ and cAMP in cells transfected with NTR-1 but not in cells transfected with NTR-2. However, in cells transfected with both NTR-1 and NTR-2 nematocin causes a decrease in cAMP levels. Therefore, nematocin may activate alternative signaling pathways in *C. elegans* depending on whether NTR-1 is expressed alone or is co-expressed with NTR-2 (41, 42).

Analysis of the expression of nematocin in *C. elegans* revealed that in both sexes it is expressed in the AFD thermosensory neurons (which mediate thermotaxis), the DVA mechanosensory neuron (which regulates locomotion and posture), neurosecretory NSM cells, AVK interneurons and the pharyngeal neuron M5. Furthermore, in males nematocin is expressed in male-specific CP motor neurons that control turning behavior during mating. Analysis of the expression of the nematocin receptor genes revealed that in both sexes *ntr-1* and *ntr-2* are expressed in partially overlapping populations of head and tail neurons. For example, *ntr-1* is expressed in the left ASE (ASEL) gustatory neuron, the chemosensory neurons ASH and ADF and the PQR tail neuron. Furthermore, in males *ntr-1* and *ntr-2* are expressed in neurons and muscles that control and enable mating behavior. Thus, *ntr-1* is expressed in hook and tail sensory neurons that sense the vulva and hermaphrodite contact and in spicule protractor muscles that are involved in sperm transfer, whereas *ntr-2* is expressed in sensory-motor tail neurons that induce spicule penetration and muscle contraction for sperm transfer and in oblique muscles that promote prolonged vulval contact (41, 42). Informed by the patterns of expression of nematocin and its two receptors, the physiological roles of nematocin signaling in gustatory processes and male mating behavior were investigated.

With respect to gustation, *C. elegans* is normally attracted to low salt concentrations as an indicator of food availability. However, if wild-type worms experience low salt concentrations in the absence of food then they exhibit a change in behavior, showing reduced attraction to or even aversion to salt at low concentrations. Interestingly, this change in behavior was found to be impaired in worms with loss of function mutations in the genes encoding nematocin or NTR-1. Therefore, it was concluded that nematocin signaling is required for normal gustatory associative learning in *C. elegans* (41).

With respect to male mating behavior, if wild-type animals are placed in an arena with food and mating partners, they usually attempt to mate with the first hermaphrodite that they make contact with. First they make one or two turns around the body of the mating partner to locate the vulva and then sperm are usually transferred successfully within 5 min. In contrast, males with a mutated nematocin gene only attempted to mate after contact with several hermaphrodites and made more turns around the hermaphrodite before locating the vulva. In addition, the mutants were defective in turning behavior, maintenance of vulval contact and transfer of sperm. More specifically, deletion of nematocin expression in the DVA mechanosensory neuron was found to cause defects in the initial contact response and efficiency in locating the vulva, but turning behavior was not affected. Furthermore, generation of mutants lacking functional nematocin receptors revealed that NTR-1 and NTR-2 have overlapping roles in mediating the effects of nematocin as a regulator of mating behavior in *C. elegans*. Thus, nematocin signaling appears to increase the effectiveness of neural circuits in generating a sequence of behaviors that result in successful sperm transfer. Furthermore, it is proposed that nematocin signaling primes “neurons in a variety of local circuits to generate a neuroethological ‘appetitive’ function in mating” (42).

Male *C. elegans* also exhibit what is referred to as long-term mate search behavior, where animals will leave a bacterial food source if hermaphrodite mating partners are not also present. Interestingly, animals with mutations in genes encoding nematocin and its receptors exhibited deficiencies in this leaving behavior, providing further evidence of the importance of nematocin signaling for reproduction-associated behavior in *C. elegans* (42). The presence of larval progeny also increases the occurrence of adult *C. elegans* leaving a food source and this appears to be triggered by larval release of pheromones. Interestingly, worms with mutations in the *nematocin* or *ntr-1* genes exhibit reduced progeny-induced food-leaving behavior, indicating that nematocin is an important regulator of this behavior. This finding is interesting because it provides evidence of an evolutionarily ancient role of VP/OT-type neuropeptide signaling in parental-offspring social behavior (77).

Although research on VP/OT-type neuropeptide signaling in nematodes has primarily focused on *C. elegans* as a model system, it is of importance to also consider the occurrence of this signaling system in other nematodes and in particular parasitic species. Interestingly, phylogenetic analyses based on analysis of genome sequence data indicate that VP/OT-type signaling has been lost in *Brugia malayi*, which causes lymphatic filariasis in humans, *Ascaris suum*, an intestinal parasite in pigs, and *Trichinella spiralis*, which causes trichinosis in humans. It has been noted that these three species all lack a free-living larval stage outside the vector or host and therefore loss of VP/OT-type signaling may be reflective of this (45).

#### Other Ecdysozoans

Analysis of genome sequence data has enabled identification of genes encoding VP/OT-type precursors in the tardigrades (water bears) *Hypsibius dujardini* and *Ramazzottius variernatus*. In both species the neuropeptide derived from the precursor protein has the predicted sequence CFVTNCPPG-NH_2_ (Table 1). Furthermore, both species have two genes encoding VP/OT-type receptors (78). However, nothing is known about the physiological roles of VP/OT-type signaling in tardigrades. These animals are well-known for their remarkable ability to survive extreme environmental conditions, including desiccation, high and low temperature and pressure, and exposure to radiation (79). In the context of the recent report of a proposed physiological role of VP/OT-type signaling in dessication resistance in ants (69), it would be interesting to determine if VP/OT-type signaling has a similar role in tardigrades. Another ecdysozoan species in which genes encoding VP/OT-type precursors/receptors have been identified is the penis worm *Priapulus caudatus* (phylum Priapulida) (47). However, as with tardigrades, nothing is known yet about the expression and function of these genes in this species.

### Spiralia

#### Mollusca

Use of antibodies to VP/OT-type peptides provided early evidence that this neuropeptide family occurs in molluscs. For example, use of immunohistochemical methods enabled identification of neurons that contain VP/OT-like peptides in the pond snail *L. stagnalis* (34) and use of radioimmunoassays and high performance liquid chromatography enabled detection of a VP-like immunoreactive peptide in extracts of ganglia from the sea slug *Aplysia californica* (33) and other gastropod species (80). However, it was not until 1987 that the molecular structure of molluscan VP/OT-type peptides was determined with the purification and sequencing of Lys-conopressin G and Arg-conopressin S from venom of the cone snails *Conus geographus* and *Conus striatus*, respectively (35).

Molecular characterization of VP/OT-type neuropeptide signaling in *L. stagnalis* was accomplished by purification of the peptide CFIRNCPKG-NH_2_ (Table 1) from neural ganglion extracts, cloning and sequencing of a cDNA and gene encoding this peptide and identification of the receptor that mediates its effects (37, 81, 82). Mapping of the expression of the VP/OT-type precursor in *L. stagnalis* revealed expression in neurons located in the anterior lobes of the cerebral ganglion in a position consistent with neurons that project into the penis nerve to innervate the penis complex and vas deferens. Accordingly, immunohistochemical analysis using antibodies to the VP/OT-type neuropeptide associated protein neurophysin revealed immunostained fibers in the penis nerve and vas deferens. Consistent with this pattern of expression, the *Lymnaea* VP/OT-type neuropeptide induces membrane depolarisation and rhythmic spiking of muscle cells isolated from the vas deferens (38) and triggers rhythmic contractions of *in vitro* preparations of the vas deferens (37). These findings indicate that VP/OT-type neuropeptide signaling has a physiological role in control of ejaculation in *L. stagnalis*. Interestingly, neurophysin-immunoreactivity was also observed in close proximity to the axons of neuroendocrine caudodorsal cells (CDC) in the cerebral ganglia, which release egg-laying peptides and control egg production and associated female reproductive behavior in *L. stagnalis*. Accordingly, *in vitro* electrophysiology experiments revealed that the *Lymnaea* VP/OT-type neuropeptide causes inhibition of CDCs by causing membrane hyperpolarisation and a reduction in spiking frequency (37). Collectively, these findings are of interest in the context of the simultaneous hermaphroditic mode of reproduction in *L. stagnalis* (83), indicating that VP/OT-type signaling in this species acts to promote male-type reproductive behavior whilst inhibiting female-type reproductive behavior. Furthermore, these findings provided some of the first evidence that VP/OT-type neuropeptides are evolutionarily ancient regulators of reproductive physiology and behavior in the Bilateria. However, VP/OT-type neuropeptide signaling is not solely involved in regulation of reproductive physiology and behavior in *L. stagnalis*. Thus, analysis of the expression of the VP/OT-type receptor in this species revealed that it is also present in the neuroendocrine light green cells and the *Lymnaea* VP/OT-type neuropeptide triggers depolarisation and spiking of these cells. Because the light green cells secrete insulin-related peptides that control somatic growth and metabolism in *L. stagnalis*, it was concluded that VP/OT-type signaling, in addition to reproductive functions, is also involved in regulation of metabolic processes in this gastropod mollusc (38).

The physiological roles of VP/OT-type neuropeptide signaling have also been investigated in detail in another gastropod—the sea slug *A. californica*. Immunohistochemical analysis of the central nervous system of this species revealed that VP-like immunoreactivity is restricted to a single neuron in the abdominal ganglion and two small neurons located bilaterally in each pedal ganglion (84). The abdominal ganglion contains neurons that control the gill-withdrawal reflex in *A. californica* and *in vitro* pharmacological studies revealed that VP/OT-type neuropeptides modulate the electrophysiological activity of identified neurons in the abdominal ganglia. For example, VP/OT-type neuropeptides decrease the spiking frequency of the gill motor neuron L7 and accordingly inhibit the gill-withdrawal reflex (33, 85–87). Furthermore, a more recent study has revealed that whilst the molluscan VP/OT-type neuropeptide conopressin-G inhibits gill motor neuron activity and gill withdrawal, it also increases the frequency of spontaneous gill movements. Interestingly, this combination of effects resembles activities associated with a food-aroused state in intact *A. californica* (85). Another reported effect of VP/OT-type neuropeptides on *A. californica* is to increase the spiking frequency of the R15 neuron (33). This neuron activates respiratory pumping and peristaltic movements of the large hermaphroditic duct during egg-laying behavior (88, 89) Thus, there is indirect evidence that VP/OT-type signaling may regulate reproductive processes in *A. californica*, although further studies are needed to specifically investigate this potential role.

VP/OT-type neuropeptide signaling has also been characterized in cephalopod molluscs, with identification of the peptide “cephalotocin” (CYFRNCPIG-NH_2_) in *Octopus vulgaris* being an important first advance (90) (Table 1). Subsequently, a second VP/OT-type peptide (“octopressin”; CFWTSCPIG-NH_2_) was identified in this species (91) (Table 1) and the genes encoding these peptides were sequenced (92). Interestingly both genes comprise single protein-coding exons, which contrasts with VP/OT-type genes in other taxa that comprise three protein-coding exons (92). The occurrence of two VP/OT-type genes/peptides in *O. vulgaris* and in other cephalopods (see below) is atypical of invertebrate species, which typically have a single VP/OT-type gene/peptide. Therefore, clade-specific gene duplication gave rise to the occurrence of two VP/OT-type genes in the cephalopod lineage, paralleling the evolution of VP and OT in the vertebrate lineage. Furthermore, two receptors that mediate effects of cephalotocin (CTR-1, CTR-2) and one receptor that mediates the effects of octopressin (OPR) have been identified in *O. vulgaris* (93, 94).

Analysis of the expression of the octopressin gene in *O. vulgaris* revealed that it is expressed in many lobes of the supraoesophageal and suboesophageal brains and in the buccal and gastric ganglia (91). Accordingly, the octopressin receptor is expressed in the brain and central administration of octopressin has been reported to evoke hyperactivity of chromatophore cells, rapid respiration and jetting of water from the siphon similar to that seen in escape behavior (43). Additionally, expression of the octopressin receptor in the buccal and gastric ganglia is indicative of a physiological role in regulation of feeding and digestion (43). The octopressin receptor is also widely expressed peripherally, including in the rectum, oviduct and efferent branchial vessel (94) and accordingly *in vitro* pharmacological tests have revealed that octopressin induces tonic contraction of preparations of the rectum, oviduct and efferent branchial vessel and rhythmic contractions of the spermatophoric gland (91). Furthermore, investigation of potential osmoregulatory roles of VP/OT-type neuropeptides in the congeneric species *Octopus ocellatus* have revealed that octopressin, but not cephalotocin, decreases hemolymph osmolality and Ca^2+^ ion concentrations, as well as urinary Na^+^ ion concentrations (95).

Analysis of the expression of the cephalotocin gene in *O. vulgaris* revealed that it is largely associated with the ventral median vasomotor lobe of the suboesophageal brain, which contains neurons that are the source of the neurosecretory system of the vena cava (91). Comparison of the expression of the cephalotocin receptor genes in *O. vulgaris* revealed that CTR1 expression is predominantly associated with the central nervous system, whereas CTR2 expression is predominantly associated with peripheral organs, including the rectum, heart, vas deferens, oviduct and branchia (94). However, cephalotocin was found to have no effect when tested on *in vitro* preparations of the rectum, oviduct, efferent branchial vessel and the spermatophoric gland (91). Further studies are now needed to gain insights into the physiological roles of cephalotocin in *O. vulgaris* and the functional significance of the occurrence of the two cephalotocin receptors.

The occurrence of two genes encoding VP/OT-type peptides has also been reported in another cephalopod species, the cuttlefish *Sepia officinalis*. Thus, one gene encodes a peptide named sepiatocin (CFWTTCPIG-NH_2_), which is most closely related to octopressin, and the second gene encodes a peptide named pro-sepiatocin (CFFRNCPPG-NH_2_), which is most closely related to cephalotocin (96) (Table 1). Immunohistochemical methods have also been used to analyse the expression of VP/OT-type neuropeptides in *S. officinalis*, but interpretation of the findings are complicated by the use of antibodies to mammalian peptides (OT and VP) and the occurrence of two VP/OT-type neuropeptides in this species (97). A more specific analysis of the expression of the sepiatocin and pro-sepiatocin genes in *S. officinalis* revealed that sepiatocin has a widespread pattern of expression in the central nervous system, consistent with the expression pattern of octopressin in *O. vulgaris*. Conversely, pro-sepiatocin expression is restricted to the supraoesophageal and suboesophageal masses of the brain, consistent with the more restricted expression of cephalotocin in *O. vulgaris* (96).

Investigation of the pharmacological actions of sepiatocin in *S. officinalis* revealed that it causes tonic contraction of the oviduct, penis and vena cava (96), consistent with the previously reported myotropic effects of octopressin in *O. vulgaris* (91). Conversely, pro-sepiatocin lacked myotropic activity in *S. officinalis* (96), consistent with previous findings for cephalotocin in *O. vulgaris* (91). Pro-sepiatocin is, however, detected in the hemolymph of *S. officinalis*, indicating that it acts as a neurohormone, with a suggested role as a regulator of reproductive processes (96). Prior to the discovery of sepiatocin and pro-sepiatocin, the homologous peptides from *O. vulgaris* (octopressin and cephalotocin) were tested for effects on long-term memory (LTM) formation of a passive avoidance task in *S. officinalis*. Cephalotocin enhanced LTM at several doses tested whereas octopressin enhanced LTM at the lowest dose tested and attenuated LTM at the highest dose tested (98). These findings provided the first evidence that VP/OT-type neuropeptides are involved in learning and memory in an invertebrate species, but further studies are now needed to gain deeper insights into the underlying neural mechanisms.

In conclusion, insights into the physiological roles of VP/OT-type neuropeptide signaling in molluscs have thus far largely been obtained from experimental studies on the gastropods *L. stagnalis* and *A. californica* and the cephalopods *O. vulgaris* and *S. officinalis*. However, analysis of transcriptome/genome sequence data has also enabled identification of genes encoding VP/OT-type precursors and/or receptors in bivalve molluscs (99, 100). For example, the precursor of a VP/OT-type neuropeptide with the sequence CFIRNCPPG-NH_2_, which is structurally very similar to pro-sepiatocin, has been identified in the scallop *Mizuhopecten yessoensis* (GenBank: OWF51696.1) (Table 1). In view of the economic importance of some bivalves as foodstuffs, investigation of the physiological roles of VP/OT-type neuropeptide signaling in these species could provide a basis for potential applications in aquaculture. With this objective in mind the physiological roles of other neuropeptides in bivalves have been investigated (101, 102), but to the best of our knowledge nothing is known about VP/OT-type neuropeptide function in bivalves.

#### Annelida

The first paper to report the identification of a VP/OT-type neuropeptide in an annelid was published in 1993. A peptide with amino acid sequence CFIRNCPKG-NH_2_ (Table 1) was purified from extracts of the central nervous system of the leech *Erpobdella octoculata* based on its immunoreactivity with antibodies to VP. Furthermore, injection of the synthetic peptide was found to cause a reduction in body mass in *E. octoculata* and it was concluded that this is due to stimulation of diuresis (103). Subsequently, the VP/OT-type peptide “annetocin” (CFVRNCPTG-NH_2_) was isolated from the earthworm *Eisenia fetida* (Table 1); however, this peptide was not isolated using antibodies but on account of its myoexcitatory activity on *in vitro* preparations of the gut (crop-gizzard complex) and nephridia (excretory organs). Thus, annetocin potentiates spontaneous rhythmic contractions of the isolated gut, potentiates pulsatile contractions of nephridia and induces pulsatile contractions in quiescent nephridia (104). Furthermore, investigation of the *in vivo* actions of annetocin in *E. fetida* revealed that it induces egg-laying related behaviors that include rotary movements, changes in body shape, mucous secretion from the clitellum and in some animals egg-laying (105). Consistent with these behavioral effects of annetocin, analysis of the expression of the annetocin precursor using mRNA *in situ* hybridization revealed that it is expressed by neurons located in the suboesophageal ganglion, which is known to be involved in regulation of reproductive behavior (106). Accordingly, immunohistochemical analysis of annetocin expression revealed a population of annetocin-immunoreactive neuronal somata in the suboesophageal ganglion and four immunoreactive neuronal somata in the cerebral ganglion. Analysis of the distribution of immunoreactive fibers revealed that they extend into the ventral nerve cord between the fourth and thirtieth segments, including the clitellum, but with a gradual reduction in the number of stained fibers proceeding posteriorly (107). Having identified and pharmacologically characterized the annetocin receptor, the expression of this receptor in *E. fetida* was investigated using mRNA *in situ* hybridization. Consistent with the effects of annetocin in inducing egg-laying related behaviors, annetocin receptor expression was found to be specifically associated with nephridia located in the clitellum region (108). Collectively, these findings indicate that a sub-population of suboesophageal neurons release annetocin from their processes in the ventral nerve cord and then annetocin binds to receptors on clitellum nephridia, which regulate production of a cocoon (108).

Interestingly, annetocin also induces egg-laying like behaviors in the leech *Whitmania pigra* (104). Accordingly, a subsequent study has shown that VP/OT-type neuropeptides induce a series of behaviors in the medicinal leech *Hirudo verbena* that closely resemble natural reproductive behavior, including twisting that aligns gonopores in preparation for copulation. Furthermore, the central pattern generator that controls this behavior was specifically localized to ganglia (M5 and M6) located in the reproductive segments of the leech (109). Annetocin was found not to induce egg-laying in the polychaete *Perinereis vancaurica* (105) but interestingly it has been reported that injection of a VP/OT-type neuropeptide in males of the polychaete *Nereis succinea* triggers swimming in tight circles and spawning (109). Analysis of the expression of the gene encoding the VP/OT-type neuropeptide CFVRNCPPG-NH_2_ in the polychaete *Platynereis dumerilii* (Table 1) revealed that it is expressed in a pair of cells adjacent to the large cilia of deep brain extraocular photoreceptor cells. Furthermore, cells expressing a ciliary-type opsin were found to be located in the same position and connected to the same axons as the cells expressing the VP/OT-type precursor. Based on these findings it was concluded that the pair of cells expressing the VP/OT-type precursor in the brain of *P. dumerilii* are extraocular photoreceptor cells. Accordingly, it was concluded that these cells may coordinate reproductive behavior with seasonal changes in light cycles (110). More recently, two receptors that are activated by the VP/OT-type neuropeptide in *P. dumerilii* have been identified (111, 112) and it would be interesting to determine the spatial and temporal patterns of expression of these receptors to gain further insights into the physiological mechanisms of VP/OT-type signaling in this important new model system in neurobiology (113).

#### Other Spiralians

A recent survey of the phylogenetic distribution of VP/OT-type signaling, based on an analysis of genome sequence data, reports that this signaling system is present in brachiopods (lamp shells) and gnathostomulids (jaw worms) but has been lost in rotifers (wheel animals) and platyhelminths (47). The loss of VP/OT-type signaling in the phylum Platyhelminthes is noteworthy given the biomedical importance of parasitic platyhelminths and extensive efforts to characterize other neuropeptide signaling systems in these animals (114–117).

### Invertebrate Deuterostomes

#### Urochordata

VP/OT-type peptides that have been identified in urochordates have a characteristic feature of this neuropeptide family in having a conserved pair of cysteines that form a disulphide bridge in the mature peptide. However, unlike the majority of VP/OT-type neuropeptides that are amidated nonapeptides, the urochordate peptides are C-terminally extended. Thus, the VP/OT-type peptide in the sea-squirt *Styela plicata* is a C-terminally amidated peptide comprising 14 residues (118) and the VP/OT-type peptide in the sea-squirt *Ciona intestinalis* (CiVP) is 13 residue peptide without C-terminal amidation (119) (Table 1). Furthermore, the receptor that mediates effects of the VP/OT-type peptide in *C. intestinalis* has been identified (119).

Using mRNA *in situ* hybridization and immunohistochemical methods, respectively, expression of the gene encoding the VP/OT-type precursor and the mature peptide was revealed in the cerebral ganglion of *S. plicata*. Interestingly, VP/OT-type precursor gene expression in the cerebral ganglion is upregulated in animals maintained in diluted seawater (60%), by comparison with animals maintained in normal (100%) or concentrated (130%) seawater. Furthermore, animals maintained in diluted seawater closed their inhalent and exhalent siphons, whereas animals in normal or concentrated seawater kept their siphons open. Consistent with these behavioral observations, *in vitro* pharmacological tests revealed that the VP/OT-type peptide causes tonic contraction of siphon muscles. Collectively, these findings indicate that the VP/OT-type peptide in *S. plicata* has a VP-like role in osmoregulatory processes, regulating water intake when animals are exposed to hypotonic seawater (118).

Consistent with findings from *S. plicata*, the VP/OT-type neuropeptide gene in *C. intestinalis* is expressed in the cerebral ganglion. Furthermore, analysis of the *C. intestinalis* VP/OT-type receptor revealed that it is expressed in the cerebral ganglion and in peripheral organs, including the digestive system, endostyle, branchia sac, heart and gonad (119). Recently, detailed functional characterization of VP/OT-type signaling in *C. intestinalis* has revealed a role in physiological mechanisms of oocyte maturation via germinal vesicle breakdown. Expression of the VP/OT-type receptor gene *CiVpr* in ovarian follicles was found to increase prior to ovulation. Furthermore, exposure of developing ovarian follicles to CiVP caused an increase in germinal vesicle breakdown and ovulation. *CiVpr* is expressed by oocytes and investigation of the mechanisms of CiVP action indicates that it causes CiVpr-mediated upregulation of the phosphorylation of extracellular signal-regulated kinase (CiErk1/2) and activation of a maturation-promoting factor, leading to oocyte maturation via germinal vesicle breakdown. Activated CiErk1/2 also induces oocyte expression of a matrix metalloproteinase (CiMMP2/9/13) and it is hypothesized that secretion of this enzyme causes digestion of collagens in the outer follicular cell layer, leading to the rupture of the follicular layer and ovulation (120).

#### Cephalochordata

Analysis of the genome sequence of the cephalochordate *Branchiostoma floridae* has enabled identification of a gene encoding a 167-residue VP/OT-type precursor (Brafl1-84802) and the neuropeptide derived from this precursor has the predicted structure CYIINCPRG-NH_2_ (22, 121) (Table 1). Two genes (154241, 134295) encoding candidate receptors for this neuropeptide have also been identified in *B. floridae* (122), but the pharmacological properties of these receptors have yet to be investigated experimentally. Furthermore, nothing is known about the expression of genes encoding the VP/OT-type precursor and receptors in *B. floridae*. This will be of interest to gain insights into the evolution of neuropeptide signaling in the phylum Chordata and comparison with VP/OT-type neuropeptide function in urochordates and vertebrates.

#### Hemichordata

Analysis of the genome sequence of the hemichordate *Saccoglossus kowalevskii* (123) has enabled identification of a gene on genomic contig 42727 that encodes a VP/OT-type precursor and the neuropeptide derived from this precursor has the predicted structure CFISDCARG-NH_2_ (121). An unusual feature of this peptide is the presence of an alanine residue at position seven, which is more typically occupied by a proline residue in members of this neuropeptide family (Table 1). Furthermore, a gene (g16853) encoding a candidate receptor for this neuropeptide has also been identified in *S. kowalevskii* (122), but the pharmacological properties of this receptor have yet to be investigated experimentally. Likewise, nothing is known about the physiological roles of VP/OT-type signaling in hemichordates, which is reflective of a general paucity of information on neuropeptide expression and function in this phylum. This will surely be a fruitful research theme for future work, both in gaining understanding of the evolution of neuropeptide function in the ambulacrarian clade of the animal kingdom and in providing insights into the functional neuroarchitecture of hemichordate nervous systems (124, 125).

#### Echinodermata

Sequencing of the genome of the sea urchin *Strongylocentrotus purpuratus* enabled the first identification of genes encoding a VP/OT-type precursor and a VP/OT-type receptor in an echinoderm (126, 127). The *S. purpuratus* VP/OT-type precursor comprises a neuropeptide (echinotocin) with the predicted structure CFISNCPKG-NH_2_ (Table 1). Echinotocin has been synthesized and tested on *in vitro* preparations of the esophagus and tube feet from the sea urchin *Echinus esculentus* and found to cause dose-dependent contraction of these neuromuscular organs (40). This effect of echinotocin in sea urchins is consistent with the myotropic actions of VP/OT-type neuropeptides in vertebrates and protostomian invertebrates (see above). However, the physiological/behavioral significance of the *in vitro* myotropic actions of echinotocin in adult sea urchins is not known. Expression of the echinotocin precursor during embryonic and larval development in *S. purpuratus* has also been investigated and interestingly peak expression (~200 transcripts per embryo) is observed at 48 h post fertilization (hpf), which corresponds to the gastrula stage when the first neuronal precursor cells are detected. However, by the early larval stage (70 hpf), when a simple nervous system is present, the level of echinotocin precursor expression is lower (~60 transcripts per larva) (128). Further studies are now needed to investigate the roles of echinotocin signaling during sea urchin development and this will be facilitated by use of gene-knockout techniques (129).

Sequencing of the neural transcriptome of the starfish *A. rubens* enabled identification of a transcript encoding a VP/OT-type precursor protein comprising the neuropeptide “asterotocin” (130) and the structure of this peptide (CLVQDCPEG-NH_2_) has been confirmed using mass spectrometry (131). An unusual characteristic of asterotocin is that it has acidic residues at positions 5 and 8, which are usually occupied by a basic or hydrophobic residue in other VP/OT-type peptides (Table 1). The asterotocin receptor has also been identified in *A. rubens* and shown to be selectively activated by asterotocin and not by mammalian VP or OT (131). To gain insights into the physiological roles of asterotocin signaling in starfish, the expression of asterotocin and the asterotocin receptor in *A. rubens* has been examined using both mRNA *in situ* hybridization and immunohistochemical techniques. Asterotocin-expressing cells and fibers are present in the central nervous system (radial nerve cords and circumoral nerve ring), the locomotory organs (tube feet), several regions of the digestive system (including the cardiac stomach) and in the body wall and associated appendages (e.g., papulae that mediate gas exchange). Asterotocin receptor-expressing cells were found to be less abundant than asterotocin-expressing cells but double labeling of asterotocin and the asterotocin receptor revealed complementary patterns of expression. Investigation of the *in vitro* pharmacological actions of asterotocin revealed that it acts as a muscle relaxant in *A. rubens*, contrasting with myotropic actions of VP/OT-type neuropeptides that have been reported in vertebrates and in other invertebrates. For example, asterotocin was found to be a potent relaxant of cardiac stomach preparations from *A. rubens*. This effect of asterotocin *in vitro* was of interest because relaxation of the cardiac stomach occurs when this organ is everted out of the mouth over the soft tissues of prey when starfish feed. Therefore, the *in vivo* effects of asterotocin were also investigated and this revealed that asterotocin is a powerful inducer of cardiac stomach eversion. Furthermore, injection of asterotocin also caused arm flexion and adoption of a body posture similar to that adopted when starfish feed on prey such as mussels. Moreover, the effect of asterotocin on body posture was so powerful that it adversely affected the ability of starfish to right themselves when upturned. Collectively, the findings of this study indicate that asterotocin signaling has an important physiological/behavioral role in triggering the unusual extra-oral feeding behavior of the starfish *A. rubens* (131). Expression of the asterotocin precursor has also been investigated in the free-swimming larvae of *A. rubens*, with transcripts detected during the brachiolaria larval stage in cells located at the tips of the brachia and associated with the adhesive disk—structures that mediate larval attachment to the substratum prior to metamorphosis (132). Therefore, it would be interesting to investigate a potential physiological role of asterotocin signaling as a regulator of larval attachment in starfish. It will also be of interest to investigate the physiological roles of asterotocin in other starfish species. Relevant to this, a gene encoding an asterotocin precursor has been identified in the crown-of-thorns starfish (COTS) *Acanthaster planci* (133) but further studies are now needed to gain insights into the physiological roles of asterotocin in this species.

Transcripts encoding VP/OT-type precursors have also been identified in the brittle star species *Ophionotus victoriae, Amphiura filiformis, and Ophiopsila aranea* and interestingly, in common with starfish, the predicted neuropeptide derived from these precursors has an acidic residue (glutamate) in the eighth position (134). For example, the VP/OT-type peptide in *O. victoriae* has the predicted structure CLVSDCPEG-NH_2_, which is identical to the *A. rubens* peptide asterotocin in all but the fourth residue (Table 1). It is noteworthy, therefore, that the unusual property of asterotocin in having acidic residues at both positions five and eight is also a feature of VP/OT-type neuropeptides in brittle stars. It is not, however, a feature of the VP/OT-type neuropeptides in other echinoderm classes (see below) and therefore it can be deduced that this structural feature evolved in a common ancestor of the asterozoan clade of extant echinoderms (135), which comprises the Asteroidea (starfish) and the Ophiuroidea (brittle stars).

The other classes of echinoderms that we have yet to consider are the Holothuroidea (sea cucumbers), which are a sister class to the Echinoidea (sea urchins) in the Echinozoan clade of extant echinoderms, and the Crinoidea (e.g., featherstars) that occupy a basal position with respect to the other extant echinoderm classes (135). VP/OT-type precursor transcript sequences have been identified in several sea cucumber species, including *Apostichopus japonicus, Holothuria glaberrima*, and *Holothuria scabra* (136, 137). The neuropeptides derived from these precursors (e.g., CFITNCPLG-NH_2_ in *A. japonicus*; “holotocin”) share more sequence similarity with the sea urchin peptide echinotocin (CFISNCPKG-NH_2_) than with asterozoan VP/OT-type neuropeptides (e.g., asterotocin) (Table 1), consistent with the sister group status of sea cucumbers (Holothuroidea) and sea urchins (Echinoidea) in the echinozoan clade of the phylum Echinodermata. Currently, nothing is known about the physiological roles of VP/OT-type neuropeptides in sea cucumbers and so this is an important objective for future studies, particularly in the context of potential applications in aquaculture of economically valuable edible species such as *A. stichopus* and *H. scabra* (136, 137).

#### Xenacoelomorpha

The phylum Xenacoelomorpha comprises three sub-phyla—xenoturbellids (e.g., *Xenoturbella bocki)*, nemertodermatids (e.g., *Nemertoderma westbladi*), and acoels (e.g., *Symsagittifera roscoffensis)—*all of which have a simple body plan without a through-gut (138–140). However, there is controversy regarding the phylogenetic position of the Xenacoelomorpha in the animal kingdom. The Nephrozoa hypothesis places xenacoelomorphs as a sister group to all other bilaterian animals (141, 142) whereas the Xenambulacraria hypothesis places xenacoelomorphs within the deuterostome clade of the Bilateria as a sister group to the Ambulacraria (Hemichordates and Echinoderms) (143). In discussing this phylum last, but after the Ambulacraria, both phylogenetic positions are compatible with the structure of this review article. Nevertheless, we note that the most recent phylogenetic analysis is supportive of the Xenambulacraria hypothesis (144) and therefore in Figure 1 the phylum Xenacoelomorpha is positioned as a sister group to the Ambulacraria.

A detailed survey of the occurrence of neuropeptide signaling systems in xenacoelomorphs has been reported recently, based on analysis of genome/transcriptome sequence data from species belonging to the three sub-phyla of the phylum (145). Genes encoding VP/OT-type precursors and receptors were identified in xenoturbellids (*X. bocki* and *Xenoturbella profunda*) and a nemertodermatid (*Ascoparia* sp.) but not in acoels. Therefore, based on the data currently available it appears that VP/OT-type signaling may have been lost in acoels. Analysis of the occurrence of other neuropeptide signaling systems in xenacoelomorphs has also revealed evidence of loss in acoels and so in this respect the absence of VP/OT-type signaling in acoels may be reflective of a more widespread simplification of neuropeptide signaling in this sub-phylum. Nevertheless, analysis of complete genome sequences in a variety of acoel species may be necessary to draw definitive conclusions regarding the loss of VP/OT-type signaling.

The sequences of the predicted neuropeptides derived from VP/OT precursors in xenoturbellids and a nemertodermatid are CLVQGCPIG-NH_2_ (in *X. bocki* and *X. profunda*) and CVIVACPRG-NH_2_ (in *Ascoparia* sp.), respectively (145) (Table 1). However, nothing is known about the expression and actions of these neuropeptides and therefore this is an important and fascinating objective for future studies.

## General Conclusions and Directions for Future Research

The evolutionary origin of VP/OT-type neuropeptide signaling in a common ancestor of the Bilateria was first determined in 1987 with the sequencing of VP/OT-type peptides in protostome invertebrate species (32, 35). This finding has been consolidated over the last two decades by genome/transcriptome sequencing and the discovery of genes/transcripts encoding VP/OT-type precursor proteins and VP/OT-type receptors in a wide range of bilaterian phyla (47, 122, 146). Invertebrates typically have one gene encoding a VP/OT-type precursor and one gene encoding a VP/OT-type receptor and it can be inferred, therefore, that this reflects the ancestral condition in Urbilateria. However, gene duplication has subsequently given rise to duplicated precursor genes and/or receptor genes in several lineages, including, for example, cephalopod molluscs, nematodes, tardigrades and vertebrates (23, 24, 41–43, 78). Furthermore, genome duplication in the vertebrate lineage has given rise to expanded families of genes encoding VP/OT-type receptors (23, 24). Conversely, in some lineages the VP/OT-type signaling system has been lost. This includes apparent instances of loss in an entire phylum (e.g., Platyhelminthes, Rotifera), although definitive proof may have to await the availability of genome sequences from extant species that are representative of all the major taxonomic branches of each phylum. Accordingly, in some phyla there are instances of clade specific loss of VP/OT-type signaling; the arthropods are example of this with loss of VP/OT-signaling in several insect orders and spiders (47). Loss of VP/OT-type signaling within specific clades of a phylum is interesting because it may ultimately provide insights into the functional significance and physiological importance of VP/OT-type signaling. Thus, if we know the physiological roles of VP/OT-type signaling in one or more clades of a phylum, we can then consider how those physiological roles of VP/OT-type signaling could be dispensed with as a consequence of gene loss in other clades. With this perspective in mind, we move on now to consider what general principles emerge from our comparative survey of what is known about VP/OT-type neuropeptide function in invertebrates.

Evidence that VP/OT-type neuropeptides regulate reproductive processes and behaviors is a prevalent finding, consistent with the roles of OT, and to a lesser extent VP, in vertebrates. Perhaps most notable are the actions of annetocin in inducing egg-laying related behaviors in annelids (105, 109), evidence that nematocin signaling is required for normal male mating behavior in the nematode *C. elegans* (42) and the recent discovery that VP/OT-type signaling regulates oocyte maturation and ovulation in the urochordate *C. intestinalis* (120). Furthermore, the discovery that VP/OT-type signaling acts differentially to control male and female reproductive systems in the mollusc *L. stagnalis* illustrates how an ancient signaling system has been adapted to regulate reproduction in the context of hermaphroditism (37, 38). In the context of these findings from a variety of phyla, it is reasonable to infer that the role of VP/OT-type signaling as a regulator of reproductive processes originated in Urbilateria. Accordingly, does VP/OT signaling regulate of reproductive processes in all extant Bilateria? In the many phyla where nothing is known about VP/OT-type neuropeptide function, this question remains to be addressed. It is noteworthy that thus far no evidence of a reproductive role has been reported in insects, although it has been reported that a VP/OT-type neuropeptide inhibits steroid release from the ovary in the blue swimming crab *P. pelagicus* (75). Therefore, further investigation of potential reproductive roles of VP/OT-type signaling in arthropods would be interesting. Furthermore, our recent report that the VP/OT-type neuropeptide asterotocin triggers fictive feeding in the starfish *A. rubens* (Phylum Echinodermata) (131) may also be relevant to reproductive behavior. Spawning in starfish is triggered by a relaxin-type neuropeptide and when starfish spawn they adopt a “humped” posture similar to that adopted when they feed on prey (147–150). Therefore, whilst there is no evidence that asterotocin can trigger the release of gametes in starfish, it is possible that asterotocin signaling is involved in neural control of postural changes associated with spawning in starfish. In this context, it would be interesting to investigate if VP/OT-type neuropeptides have effects on behavior associated with reproduction in other echinoderms (e.g., sea urchins, sea cucumbers).

A second general theme that emerges from comparative analysis of VP/OT-type neuropeptide function in invertebrates are roles associated with water/salt homeostasis and excretory organs, consistent with the actions of VP as an antidiuretic in vertebrates. This includes evidence from the urochordate *S. plicata*, where VP/OT-type signaling appears to be involved in preventing intake of dilute seawater by causing closure of inhalent/exhalent siphons (118). Furthermore, it is noteworthy that annetocin has myoexcitatory effects on excretory organs (nephridia) in the earthworm *E. foetida*, which is important in a reproductive context where specialized nephridia in the clittelar region secrete a cocoon during egg-laying (104). However, this may be reflective of a more general role of VP/OT-type signaling in regulation of nephridial function in annelids and therefore it would be interesting to investigate the actions of VP/OT-type neuropeptides on species from other annelid clades [e.g., in the ragworm *P. dumerilii*; (151)]. Investigation of VP/OT-type neuropeptide function in insects has revealed what appear to conflicting actions with respect to water preservation. Thus, experimental studies on locusts and beetles have revealed a diuretic effect (i.e., water loss) (54, 64) whilst more recent studies on ants have provided evidence that the VP/OT-type neuropeptide inotocin promotes dessication resistance (i.e., water preservation) by stimulating production of cuticular hydrocarbons by oenocytes (69). In this context, it would be timely to revisit investigation VP/OT-type neuropeptide function in the locust *L. migratoria*, which as a large insect is an attractive experimental model for physiological studies. Thus, building upon previous anatomical and physiological studies (52, 56), it would be interesting to determine where the inotocin receptor is expressed in locusts as this would provide a basis for an integrative approach to analysis of the physiological roles of VP/OT-type neuropeptide signaling in this species. Furthermore, one of the most intriguing characteristics of VP/OT-type neuropeptide signaling in insects is the highly conserved expression of inotocin in a pair of neurons located in the suboesophageal ganglion, although in some species additional inotocin-expressing neurons are present in the brain (53, 64, 68, 69). This highly restricted pattern of neuronal expression may, at least in part, explain why loss of VP/OT-type neuropeptide signaling has occurred in some insect lineages (including *Drosophila*). If further advances are made in our understanding of the physiological roles of VP/OT-type signaling in insects that have retained this system, it may be possible to provide evolutionary, ecological and physiological explanations for its loss in some insect taxa.

A third emergent theme is an association of VP/OT-type neuropeptide signaling with extra-ocular photoreceptive systems and therefore by inference a potential role in circadian and/or circalunar physiological/behavioral rhythmicity. Evidence of this from invertebrates was first obtained from experimental characterization of neural inputs to suboesophageal VPLI neurons in the locust *L. migratoria* (56, 57). However, evidence of a phylogenetically more widespread association with extra-ocular photoreceptors was obtained subsequently from the annelid *P. dumerilii*, where it was concluded that suboesophageal neurons expressing the VP/OT-type neuropeptide in this species are in fact extraocular receptors themselves (110). The association of VP/OT-type neuropeptide expressing neurons with extraocular photoreception in these two invertebrate species is consistent with findings from mammals, where elevated pituitary release of OT and VP occurs during the hours of sleep (152). However, further experimental studies are now needed to investigate in more detail a proposed association of VP/OT-type signaling with circadian and/or circalunar physiological/behavioral rhythmicity in insects, annelids and/or other invertebrates.

A fourth theme that emerges from comparative studies is the role of VP/OT-type signaling in regulation of feeding, with the inhibitory effect of OT-type neuropeptides on food intake in mammals and other vertebrates (20, 27) providing a basis for investigation of feeding-related roles in invertebrates. Our discovery that asterotocin triggers fictive feeding in the starfish *A. rubens* is perhaps the most striking evidence obtained from invertebrates, although it is of course noteworthy that actions of asterotocin are indicative of an orexigenic action that contrasts with the anorexigenic action of oxytocin in mammals. Indirect evidence of VP/OT-type signaling regulating feeding and/or digestion includes the expression of VP/OT-type receptors in the digestive system and regions of nervous system that control feeding and/or digestive processes in molluscs (91), annelids (104), nematodes (41) and crustaceans (73, 74) and changes in inotocin precursor expression following a starvation period in ants (68). However, further studies are now needed to investigate in more detail the roles of VP/OT-type neuropeptides in feeding/digestion in these and other invertebrate taxa.

In summary, it is clear that in the context of comparative analysis of neuropeptide function in the Bilateria, research on VP/OT-type neuropeptide signaling has been remarkably fruitful. This may in part reflect the high level of evolutionary conservation of VP/OT-type neuropeptide/precursor structure in the Bilateria and the ease with which VP/OT-type neuropeptides can be identified in different taxa. However, it is also noteworthy that VP/OT-type neuropeptides have quite striking effects of reproduction and/or feeding related behaviors in invertebrates, which has facilitated and encouraged detailed experimental analysis of VP/OT-type signaling in a wide range of taxa. But there is still much to be learnt about the physiological roles of VP/OT-type neuropeptide signaling invertebrates if we are to achieve a level of understanding that would facilitate reconstruction of the evolution of VP/OT-type neuropeptide function in the Bilateria. Neuropeptide signaling systems operate in the context of neuronal circuits and adaptive evolutionary changes in the configuration of those circuits (153). To understand the evolution of VP/OT-type neuropeptide function it may therefore be necessary to not only determine the actions of the peptides but also to characterize the transcriptomic/proteomic/metabolomic profiles of cells expressing VP/OT-type precursors and/or VP/OT-type receptors within the framework of anatomically and functionally identified neuronal connectivities. This will not be feasible in all taxa but this should not deter comparative physiologists from taxa that were intractable prior to the genome/transcriptome-sequencing era. Investigation of the effects VP/OT-type neuropeptides in the huge variety of invertebrates that remain to be studied is sure to reveal actions that are both amazing and informative. So let this be a mission that could be celebrated in 2055, the 100th anniversary of the Nobel Prize award for chemistry to Vincent du Vigneaud.

## Author Contributions

All authors listed have made a substantial, direct and intellectual contribution to the work, and approved it for publication.

## Conflict of Interest

The authors declare that the research was conducted in the absence of any commercial or financial relationships that could be construed as a potential conflict of interest.
